# Neuronal mechanism for neuropathic pain

**DOI:** 10.1186/1744-8069-3-14

**Published:** 2007-06-06

**Authors:** Min Zhuo

**Affiliations:** 1 Department of Physiology, Faculty of Medicine, University of Toronto Centre for the study of Pain, University of Toronto, 1 King's College Circle, Toronto, Ontario M5S 1A8, Canada

## Abstract

Among different forms of persistent pain, neuropathic pain presents as a most difficult task for basic researchers and clinicians. Despite recent rapid development of neuroscience and modern techniques related to drug discovery, effective drugs based on clear basic mechanisms are still lacking. Here, I will review the basic neuronal mechanisms that maybe involved in neuropathic pain. I will present the problem of neuropathic pain as a rather difficult task for neuroscientists, and we may have to wait for a long time before we fully understand how brain encode, store, and retrieve painful information after the injury. I propose that neuropathic pain as a major brain disease, rather being a clinic problem due to peripheral injury.

## Background

Pleasure and pain are two major emotions of animals and humans. While the pleasure from different activities is what animals and human are seeking daily, both animals and humans avoid pain. Pain is divided into two major groups: physiological pain and pathological pain. Physiological pain is an important physiological function for survival. Depending on pain experience, animals and humans gain knowledge of potential dangerous stimuli in the environment, and pain-related unpleasantness help to form long-term avoidance memory in order to protect themselves. Although animals have the capability to enhance their sensitivity as well as motor responses to subsequent noxious stimuli, animals' ability to distinguish pain from other sensation is intact, at least not permanently altered.

Unlike physiological pain, pathological pain only happen after injury (e.g., tissue or nerve injury), and is not the result of repetitive application of physiological pain. Long-term changes are likely to occur after injury, both peripherally and centrally. Consequently, the injury and injury-related areas undergo long-term plastic changes, and pain sensation is significantly enhanced (hyperalgesia) or non-noxious stimuli cause pain (allodynia). It should be pointed out that allodynia is one of the major problems in pathological pain. Because it is induced by non-noxious stimuli, it is mostly likely that central plastic changes play important roles.

Thus, pathological pain is likely a result of long-term plastic changes along somatosensory pathways, from the periphery to cortex. Due to long-term plastic changes in central regions, pain specificity is lost at the first synapses of the somatosensory pathway, at least from areas where allodynia was reported. Here I will review recent progress related to neuronal mechanism for neuropathic pain, a form of persistent pain resistant to conventional treatment. I will focus on central plasticity, in particular the forebrain areas that are critical for the processing pain and pain-related emotional responses. Readers are referred to other reviews published in this series for other areas and mechanisms, such as the spinal cord [1] and amygdala.

## Activation of spinal neurons: synaptic events in physiological pain

Peripheral noxious stimuli activate peripheral nociceptive transducer receptor and/or ion channels, and cause membrane depolarization in sensory dorsal root ganglion (DRG) cells. Transducer proteins include a family of proteins, including TRPV1-4, TRPM8, ATP receptor etc [2,3]. It becomes clear that no simple protein or gene is responsible for a specific sensory process, for example, heat pain or cold sensation. Thus, a peripheral sensory protein may contribute multiple sensory processes, such as heat, cold, itch and touch. Under physiological conditions, noxious stimuli stimulate both non-noxious as well as nociceptive fibers. It is almost impossible to deliver a selective noxious stimuli without activate some form of non-nociceptive receptors. In pathological pain condition, typical allodynia triggered by non-noxious stimulation is also unlikely due to selective activation of nociceptive fibers. It is safe to say that each sensory modality or sensation is a function of a specially organized neuronal circuit and network, from the periphery to the cortex with some of key proteins playing major roles.

**Figure 1:**
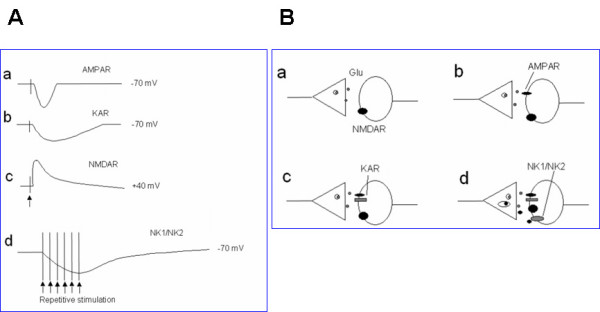
**Excitatory sensory synapses contribute to pain transmission in the spinal dorsal horn and ACC**. A. Synaptic currents recorded at resting membrane potentials are mostly mediated by AMPA receptors (a), some synaptic currents at dorsal horn neurons receiving high-threshold inputs are mediated by KA receptors (b). In young and adult dorsal horn neurons, some sensory synapses are 'silent' and containing only functional NMDA receptors (c). These pure NMDA synapses can be revealed when cells are hold at positive 40 mV potentials. In adult dorsal horn neurons, some pure NMDA receptor synapses can even be detected at the resting membrane potentials. When a train of stimulation is applied, neuropeptide mediated responses are recruited. Both postsynaptic NK1 and NK2 receptors contribute to substance P(SP)- and neurokinin A- (NKA) mediated excitatory postsynaptic currents. In ACC neurons, KA receptor mediated responses are detected with stimulation at greater intensities. KA receptors do not contribute to mEPSCs, indicating that KA receptors are not co-expressed with AMPA receptors in all synapses. B. Models for glutamate-containing and glutamate- and neuropeptide-mixed sensory synapses in the spinal cord dorsal horn and possible ACC neurons. At least four different synapses are found: (a) synapses receiving low-threshold sensory inputs contain only postsynaptic NMDA receptors; (b) synapses receiving low-threshold sensory inputs contain both AMPA and NMDA receptors; (c) synapses receiving both low- and high-threshold sensory inputs contain postsynaptic AMPA, KA and NMDA receptors; (d) synapses receiving low- and high-threshold sensory inputs contain AMPA, KA and NMDA receptors as well as peptidergic NK1 and NK2 receptors.

Primary afferent fibers form synapses with dorsal horn sensory neurons in the spinal cord. Some of these dorsal horn neurons send ascending projecting fibers and make synapses with neurons located at supraspinal sites, such as the thalamic nuclei. These ascending pathways are important for conveying sensory information from the periphery to the brain. Glutamate is a major neurotransmitter between primary afferent fibers and dorsal horn neurons [4,5] and postsynaptic responses are mainly mediated by glutamate α-amino-3-hydroxy-5-methyl-4-isoxalepropionate (AMPA) and kainate (KA) receptors [4-7] (see fig 1). Glutamatergic synapses are heterogeneous in the spinal dorsal horn [4,6,8,9], and at least three different types of glutamatergic synapses have been reported [4,8,10]. In some synapses receiving low threshold sensory inputs, only postsynaptic NMDA receptors are found. In synapses receiving low- or moderate intensity of sensory inputs, only AMPA receptors are detected, while in synapses that receiving high threshold inputs, both glutamatergic AMPA and KA receptor are reported.

In addition to glutamate, several neuropeptides including substance P (SP) are thought to act as sensory transmitters (see fig 1). Whole-cell patch-clamp recordings reveal relatively faster SP and neurokinin A-mediated synaptic currents in synapses between primary afferent fibers and EPSCs in case of burst activity allow SP-mediated responses affect the excitability of spinal dorsal horn neurons [11]. Together with glutamate-mediated synaptic responses, these neuropeptide-mediated EPSCs may cause dorsal horn neurons to fire action potentials at a high frequency for a long period of time. The combination of glutamate- and neuropeptide-mediated EPSCs allow nociceptive information to be conveyed from the periphery to the central nervous system (see fig 1).

## Anterior cingulate cortex (ACC): a major cortical area for pain

ACC is a major forebrain area for pain-related perception. The ACC contains layers of pyramidal cells and local interneurons. The layers II-III contain mainly pyramidal cells and neurons in layer II-III receive sensory inputs from the medial thalamus, a key relay nucleus for somatosensory information including pain [12-14]. Neurons in layer V are larger than cells in layers II-III and VI, and also receive sensory inputs including noxious information. ACC neurons form interconnection with the other ACC neurons in the opposite side of the hemisphere through callosal projecting fibers, as well as other cortical areas at the same side and opposite side of the brains.

Most ACC neurons respond to both noxious and non-noxious stimuli. Many non-pyramidal cells are inhibitory neurons that contain GABA and/or neuropeptides. It typically shows reduced spike responses to peripheral noxious stimuli, while pyramidal cells show excitatory or increased spike responses. It is important to note that while the soma of the pyramidal cells may be strictly located at the layer V, its distal branches can extend to other layers of the ACC such as layer I. Thus, the evoked responses recorded from the layer V cells maybe contain synaptic responses occurring at outer layers of the ACC. Within the local circuits, inhibitory neurons often receive innervations from the pyramidal cells (glutamatergic), then release GABA onto the perisomatic region of the pyramidal cells. Based on differences in their morphology and the pattern of spike activities, pyramidal and inhibitory neurons can be identified electrophysiologically in brain slice preparations [15,16].

## Neuronal output from the ACC

Pyramidal cells in the layer V project to subcortical structures such as the hypothalamus and periaqueductal gray (PAG), may contribute to descending modulation of spinal sensory transmission [for example, see [17]]. ACC neurons also form interconnections with neurons in the amygdala, a structure critical for emotional fear and anxiety. This anatomic connection suggests possible critical roles of ACC in emotional fear, anxiety and depression. Furthermore, ACC neurons project to motor cortex to generate motor responses such as emotional vocalization. Thus, it is likely that ACC may serve as a key area for pain interacting with other cognitive functions. For example, inflammation leads to changes in ACC synaptic plasticity, and related loss of trace fear memory [18]. Behavioral ***in vivo ***studies show that ACC is important for pain processing. Lesions of the ACC significantly increased acute nociceptive responses, and formalin injection induced aversive memory behaviors [19,20]. In patients with frontal lobotomies or cingulotomies, the unpleasantness of pain is abolished [see [21] for review]. Neuroimaging studies further confirm these observations and show that the ACC, together with other cortical structures, are activated by acute noxious stimuli [22-25]. Recent studies from healthy humans or patients suggest that ACC activity is also related to the empathy of pain, social exclusion/pain, chronic migraine as well as hypothesized pain [26-31]. Stimulation of ACC neurons by delivering electrical currents or local glutamate microinjection caused fear memory or trigger aversive behaviors, indicating that stimulation of the ACC is painful, fearful or aversive [29,32,33].

## Insular cortex: a an area remained to be explored at molecular level

Similar to the ACC, insular cortex has been also reported to play roles in pain, although most of data are imaging data in human patients [22-25,34]. Local manipulations that enhancing GABA functions in the insular cortex produced long-lasting analgesic effects [34]. Future studies are clearly needed for detailed synaptic analyses and anatomy of insular cortex.

## Activity-dependent plasticity under experimental conditions

**Figure 2:**
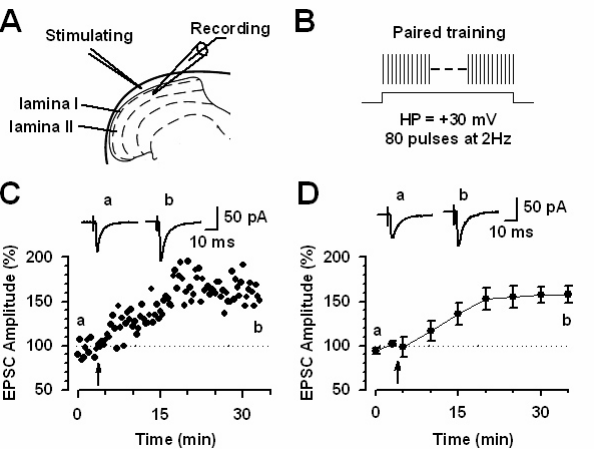
**Long-term potentiation (LTP) of sensory excitatory synapses in adult mouse spinal cord**. A. Diagram of a spinal slice showing the placement of a whole-cell patch-clamp recording and a stimulation electrodes in the superficial dorsal horn of the adult mouse spinal cord. B. Schematic illustrating the induction protocol consisting 80 pulses at 2 Hz while holding at +30 mV (paired training). C. A example of spinal LTP induced by paired training protocol in a superficial dorsal horn neuron. D. Summary result of the LTP experiments under control conditions (*n *= 9 neurons). EPSC responses were averaged over five-minute intervals [Modified from 40].

Studies of long-term potentiation (LTP) in spinal dorsal horn neurons draw much attention, because it is believed that potentiation of sensory responses after injury may explain chronic pain [1,35-37]. While it has been consistently demonstrated that spike responses of dorsal horn neurons to peripheral stimulation are enhanced after the injury [see [36]], it remains to be investigated if enhanced spike responses are simply due to enhanced synaptic transmission between the DRG cells and dorsal horn neurons. Unlike synapses in other areas such as hippocampus, synaptic potentiation in the spinal dorsal horn neurons is not induced by strong tetanic stimulation [38]. Recent studies further show that LTP only happen in some of spinal projecting cells [39]. In spinal cord dorsal horn neurons that did not express SP receptors did not undergo potentiation. Furthermore, activation of NK1 receptors or NMDA receptors is required for LTP. However, in other areas of the brain, there is no requirement of SP for the induction of NMDA receptor dependent LTP [39]. Using the classic pairing protocol, Wei et al. [40] reported that LTP can be induced in dorsal horn neurons in adult mouse dorsal horn neurons (see fig 2). One key experiment is needed to directly demonstrate that neurons receiving nociceptive inputs undergo LTP in the spinal cord.

In addition to LTP, long-term facilitation induced by neurotransmitter such as serotonin (5-HT) has been reported [4,7]. At the molecular level, postsynaptic glutamate receptors are organized into the place through a family of proteins containing PDZ domains [see [10] for review]. Furthermore, these postsynaptic protein-protein interactions are very dynamic and can be involved in the clustering, removal or insertion of postsynaptic receptors [7], providing a novel and efficient way to regulate synaptic strength. Protein-protein interactions contribute to switching the phenotype of dorsal horn sensory synapses, i.e., changing silent synapses into functional synapses [7].

## Excitatory synaptic transmission and plasticity in the ACC

Glutamate is the major fast excitatory transmitter in the ACC [41,42]. Different types of glutamate receptors, including AMPA, KA, NMDA and metabotropic receptors (mGluRs) are distributed in the ACC (see fig 1). Fast synaptic responses induced by local stimulation or stimulation of thalamocortical projection pathways are mostly mediated by AMPA receptors. Recent studies using whole-cell patch-clamp recordings from genetically modified mice show that postsynaptic KA receptors contribute to fast synaptic transmission in pyramidal neurons in the ACC [42]. Moreover, GluR5 containing KA receptors also modulate GABAergic transmission in the ACC [43]. Single-shock stimulation could induce small KA receptor-mediated excitatory postsynaptic currents (KA EPSCs). Genetic deletion of the GluR6 or GluR5 subunit significantly reduced, and GluR5 and 6 double knockout completely abolished, KA EPSCs and KA-activated currents in ACC pyramidal neurons [42]. We believe that KA receptors in the ACC may also contribute to pain and its related emotion such as anxiety [44,45].

**Figure 3:**
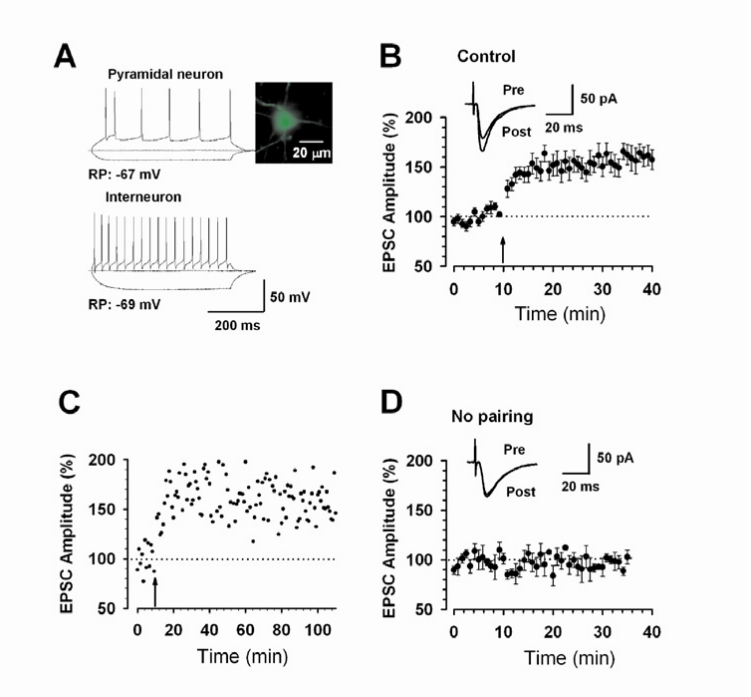
**Long-term potentiation of sensory excitatory synapses in adult mouse ACC**. A. Current-clamp recordings to identify pyramidal neurons (upper) and interneurons (bottom) by current injections of -100, 0, and 100 pA. A labeled pyramid-like neuron was shown on the top right. RP: resting membrane potential. B. LTP was induced in pyramidal neurons (n = 15) in adult ACC by the pairing training protocol (indicated by an arrow). The insets show averages of 6 EPSCs 5 min before and 25 min after the pairing training (arrow). The dashed line indicates the mean basal synaptic responses. C. An example showing the long-lasting synaptic potentiation. Pairing training is indicated by an arrow. D. Basic synaptic transmission showing no change during recording without applying pairing training. The insets show averages of 6 EPSCs at the time points of 5 (pre) and 35 (post) min during the recording [Modified from 15].

In the ACC, the induction of LTP by different protocols is mostly NMDA receptor dependent. NMDA receptor containing NR2A or NR2B subunits contribute to most of NMDA receptor currents [15]. Bath application of a NR2A antagonist NVP-AAM077 and NR2B antagonist ifenprodil/Ro compounds produce almost completely blockade of NMDA receptor mediated EPSCs. Application of NR2A or NR2B antagonist reduces LTP, without complete abolishment of LTP. LTP is only abolished after the co-application of both inhibitors [15]. It is noted that LTP induced by spike-timing protocol seems to more sensitive to NMDA NR2B blockade as compared with effects on LTP induced by pairing training protocol [15]. In addition to NMDA receptors, L-type voltage gated calcium channels (L-VDCCs) are required for inducing LTP [46] when LTP is induced by theta-burst stimulation (TBS) in field recording conditions. Therefore, it is likely that ACC LTP induced by different protocols may mimic different physiological/pathological conditions by distinct signaling pathways (see fig 3).

**Figure 4:**
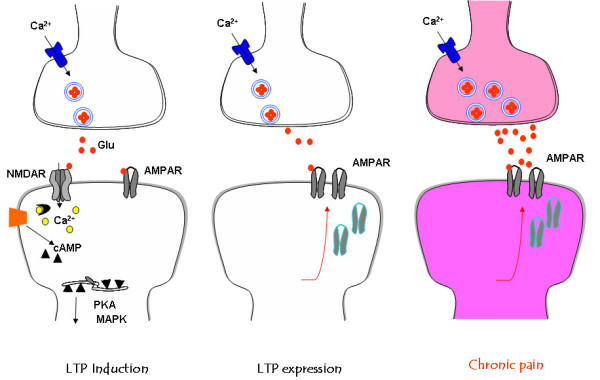
**Model of signaling pathways for LTP and central plasticity caused by injuries**. A. In the ACC, activity triggers release excitatory neurotransmitter glutamate (Glu: filled circles). Activation of glutamate NMDA receptors leads to an increase in postsynaptic Ca^2+ ^in dendritic spines. Ca^2+ ^serves as an important intracellular signal for triggering a series of biochemical events that contribute to the expression of LTP. Neural Ca^2+ ^binds to CaM and leads to activation of calcium-stimulated ACs, including AC1 and AC8 and Ca^2+^/CaM dependent protein kinases (PKC, CaMKII and CaMKIV). The Ca/CaM dependent protein kinases phorsphoryalte glutamate AMPA receptors, increasing their sensitivity to glutamate. B. In the ACC synapses, the possible postsynaptic trafficking of AMPA GluR1 receptors contributes to synaptic potentiation. C. Inflammatory or nerve injuries cause both presynaptic and postsynaptic changes in the ACC synapses. Enhanced release of glutamate as well as postsynaptic changes in AMPA receptor mediated responses contribute to enhanced noxious sensory information with in the brain, a possible cellular mechanism for persistent pain.

Activation of glutamate NMDA receptors and/or L-VDCCs leads to an increase in postsynaptic Ca^2+ ^in dendritic spines. Ca^2+ ^serves as an important intracellular signal for triggering a series of biochemical events that contribute to the expression of LTP (see fig 4). Calcium binds to CaM and leads to activation of calcium-stimulated signaling pathways [47]. Furthermore, postsynaptic injection of BAPTA completely blocked the induction of LTP, indicating the importance of elevated postsynaptic Ca^2+ ^concentrations [15]. A recent work using electroporation of mutant CaM in the ACC suggest that Calcium binding sites of CaM is critical for the induction of cingulate LTP [47]. AC1 and AC8 are two AC subtypes that respond positively to calcium-CaM [48]. As compared with AC8, AC1 is more sensitive to calcium increase. In the ACC, AC1 is highly expressed in cingulate neurons located in most of layers [49]. AC1 is selective for plastic changes and gene deletion of AC1 does not affect basal glutamate transmission in the ACC. By contrast, LTP induced by TBS or pairing stimulation are abolished in cingulate pyramidal cells [46]. Whole-cell patch-clamp recording also revealed that AC1 activity is required for the induction of LTP in ACC pyramidal cells. By using chemical design and biochemical screening, several selective inhibitors of AC1 has been identified. Consistently, pharmacological inhibition of AC1 in ACC neurons abolished LTP induced by pairing training (Zhuo, unpublished data).

Among these possibilities, we have recently investigated the roles of GluR1 and GluR2/3 using genetic and pharmacological approaches. We found that GluR1 subunit C-terminal peptide analog, Pep1-TGL, blocked the induction of cingulate LTP [50]. Thus, in the ACC, the interaction between the C-terminus of GluR1 and PDZ domain proteins is required for the induction of LTP. Synaptic delivery of the GluR1 subunit from extrasynaptic sites is the key mechanism underlying synaptic plasticity [51,52] and GluR1-PDZ interactions play a critical intermediate in this plasticity. Our pharmacological experiments show that the application of Philanthotoxin-433 (PhTx) 5 min after paired training reduced to synaptic potentiation, while PhTx had no effect on basal responses. Therefore, we believe that Ca^2+^-permeable GluR2-lacking receptors contribute to the maintenance of LTP and are necessary for subsequent LTP stabilization. Although our data did not provide direct evidence for the synaptic trafficking or insertion of GluR1 receptors at postsynaptic membrane, the present findings suggest selective contribution of AMPA subtype receptors to cingulate LTP (see fig 4).

## LTP in the insular cortex

LTP in the insular cortex has been reported in brain slice preparation [53] and in vivo freely moving rats [54]. Theta-burst stimulation produced long-lasting enhancement of synaptic responses in the insulate cortex of adult mice. Ca-CaM-activated CaMKIV is required for the synaptic potentiation [53]. More studies are needed for synaptic mechanism for insular LTP.

## Long-term plastic changes after nerve injury

One important question related to ACC plasticity is whether injury causes prolonged or long-term changes in synaptic transmission in the ACC in whole animals. To test this question, we first measured synaptic responses to peripheral electrical shocks. The field EPSPs recorded from the ACC was obviously polysynaptic in nature, likely involving at least primary afferent fibers and spinothalamic and thalamocortical tracts. To detect central plastic changes, we performed amputation at the hindpaw contralateral to the one to which stimulation was delivered. Interestingly, after amputation of a central digit of the hindpaw, we observed a rapid enhancement of sensory responses to peripheral electrical shocks delivered to the normal hindpaw. The potentiation was long-lasting; evoked responses remained enhanced for at least 120 min [55]. We also observed a long-lasting potentiation of field EPSPs after amputation that lasted for at least 90 min [55]. ***In vivo ***intracellular recordings from cortical pyramidal cells confirm the long-term changes in excitatory synaptic transmission [56]. These ***in vivo ***results provide direct evidence that excitatory synaptic transmission within the ACC undergo long-term potentiation after peripheral injury. In support of plastic changes in the ACC after injury, activity-dependent immediate early genes, such as c-fos, Egr1, adenosine 3',5'-monophosphate response element binding protein (CREB) are activated in the ACC neurons after tissue inflammation or amputation [41,57]. Furthermore, these plastic changes persist for a long period of time, from hours to days. Studies using AC1&AC8 double knockout or NR2B overexpression mice show that NMDA receptors, AC1 and AC8 contribute to activation of immediate early genes by injury [49,57] (see fig 4).

## Loss of in vitro ACC long-term depression (LTD) after injury

In parallel with these dramatic changes in gene expression, synaptic plasticity recorded from in vitro ACC slices is also altered. In ACC slices of animals with amputation, the same repetitive stimulation produced less or no LTD. The loss of LTD is regionally selective, and no change was found in other cortical areas [41]. One possible physiological mechanism for LTD in the ACC is to serve as an autoregulatory mechanism. LTD induced during low-frequency repetitive stimulation may help to maintain appropriate neuronal activity within the ACC by reducing synaptic transmission. In amputated or injured animals, the loss of autoregulation of synaptic tone may lead to overexcitation in the ACC neurons and contribute to enhancement of pain or unpleasantness related to the injury (see fig 4).

## LTP may not *completely *mimic synaptic mechanisms of persistent pain

LTP has been proposed as a cellular mechanism for learning and memory. Although the link of LTP with behavioral memory is still remained unclear, many neuroscientists are intensely focusing on presynaptic and postsynaptic contribution to the LTP. It had to be pointed that different forms of LTP are likely occurring using various induction protocols. Thus, it is critical to ask what form of learning-related mechanisms can be mimicked by slice preparations.

ACC LTP is induced by different protocols. Different receptors may be involved. For example, in whole-cell patch recording, pairing protocol induced LTP is pure NMDA receptor dependent, and activation of L-VDCCs is not required However, under the field recording condition, LTP induced by the theta burst stimulation is reduced by L-VDCC antagonist [46]. Thus, it is wrong to conclude that pairing LTP is more appropriate than TBS for studying pain-relate plasticity. In fact, both presynaptic and postsynaptic mechanisms are found in the ACC glutamatergic synapses after injury [[16,18], unpublished data, [56]]. These findings suggest that it may require different forms of LTP to mimic the pathological mechanisms of persistent pain. It is thus easy to believe that different forms of memory are likely mediated by different mechanisms.

## ACC and endogenous descending modulation

In addition to descending inhibition, descending excitatory or facilitatory influences from the brainstem or forebrains have been characterized [17,58-62]. Biphasic modulation of spinal nociceptive transmission from the rostroventromedial medulla (RVM), perhaps reflecting the different types of neurons identified in this area, offer fine regulation of spinal sensory thresholds and responses. While descending inhibition is primarily involved in regulating suprathreshold responses to noxious stimuli, descending facilitation reduces the neuronal threshold to nociceptive stimulation [58-62].

Descending facilitation has a general impact on spinal sensory transmission, inducing sensory inputs from cutaneous and visceral organs [63,64]. Descending facilitation can be activated under physiological conditions, and one physiological function of descending facilitation is to enhance animals' ability to detect potential dangerous signals in the environment. Indeed, neurons in the RVM not only respond to noxious stimuli, but also show 'learning'-type changes during repetitive noxious stimuli. More importantly, RVM neurons can undergo plastic changes during and after tissue injury and inflammation. Descending facilitation is likely activated after the injury, contributing to secondary hyperalgesia [65,66]. Blocking descending facilitation, by lesion of the RVM or spinal blockade of serotonin receptors, is antinociceptive [67-69]. The descending facilitatory system therefore serves as a double-edged sword in the central nervous system. On one hand, it allows neurons in different parts of the brain to communicate with each other and enhances sensitivity to potentially dangerous signals; on the other hand, prolonged facilitation of spinal nociceptive transmission after injury speeds up central plastic changes related to chronic pain.

## Positive feedback controls at synaptic and systemic levels

Understanding physiological mechanisms of chronic pain will not be revealed if we only learn mechanisms at the level of individual synapses. Changes in individual synapses can lead to alterations of neuronal network functions related to pain transmission and modulation. A ***positive feedback control ***has been proposed to serve as the key pathological mechanism for chronic pain [21]. Positive enhancement occurs not only at single synapses, but also between multiple neuronal synapses at different parts of the brain. Several mechanisms may contribute to synaptic enhancement:

(1) postsynaptic regulation of glutamate receptors, including phosphorylation and dephosphorylation;

(2) recruitment of functional glutamate receptors (for example, in spinal dorsal horn neurons, recruitment of postsynaptic functional AMPA receptors);

 (3) presynaptic enhancement of glutamate release;

(4) structural changes in synapses.

**Figure 5:**
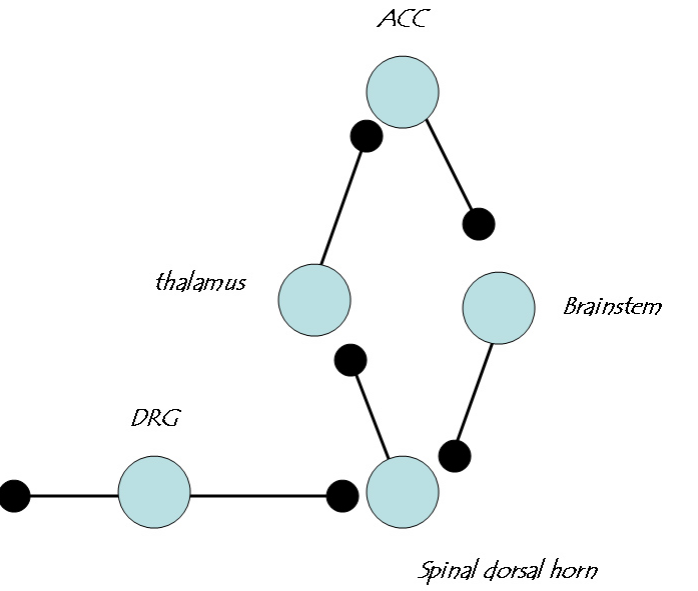
***Positive feedback control *as a key central mechanism for persistent pain**. Sensory inputs inducing painful stimuli enter the brain through three major synaptic relays, including the dorsal horn (DH), thalamus and anterior cingulate cortex (ACC). At each synaptic relay, glutamate is the major fast excitatory transmitter. While AMPA and KA receptors mediate most of the synaptic response at resting conditions, NMDA receptors serve as a coincidence detector to enhance synaptic responses in an activity-dependent manner. Long-lasting potentiation is likely to occur at each sensory synapse. Within the ACCs, cortical connections may also undergo plastic changes, and may serve as highest central loci to store unpleasantness or pain. In addition, dorsal horn sensory synapses receive heterosynaptic facilitatory modulation from supraspinal structures, including the forebrain and brainstem. The RVM in the brainstem is likely to serve as a final relay for this descending facilitatory or excitatory modulation. Both homosynaptic and heterosynaptic enhancement will lead central sensory neurons to an enhanced excitatory status, so that a gentle trigger (for example, allodynia) or stimulation can cause massive firing of action potentials and thus cause pain. In the case of central pain, spontaneous activity of neurons in the network itself can also lead to action potential firing and pain.

At network levels, heterosynaptic facilitation or dis-inhibition can lead to enhancement as well. It is well documented that dorsal horn neurons receive descending facilitatory modulation from the brainstem neurons. Activation of supraspinal structures including ACC neurons can also facilitate spinal responses [17,62] and triggers long-term fear memory [33]. The consequence of this ***positive feedback control ***will lead central neurons to a much enhanced and overexcited status; a weak input will lead to significantly greater neuronal action potentials. Such a mechanism most likely contributes to several chronic pain related states, such as allodynia and central pain (see fig 5).

## Future directions

Revealing the causal relationships between synaptic plasticity with behavioral persistent pain is just the beginning to understanding of chronic pain. To erase or reset altered synapses after the injury may present most difficult task for neuroscientists and pain researchers. Changes in glutamate AMPA receptors or so called postsynaptic mechanisms of LTP, may only represent early changes in central synapses. The integration of individual synaptic changes to neuronal action potentials is still a mystery. In addition to changes in glutamaterigc synapses, changes in inhibitory synapses, glial cells have been reported [70,71]. Future studies to reveal the excitation(E)/inhibition (I) relationship and how changes in glial cells affect brain process of sensory information are clearly needed.

## References

[B1] Sandkuhler J (2007). Understanding LTP in pain pathways. Mol Pain.

[B2] Lumpkin EA, Caterina MJ (2007). Mechanisms of sensory transduction in the skin. Nature.

[B3] McKemy DD (2005). How cold is it? TRPM8 and TRPA1 in the molecular logic of cold sensation. Mol Pain.

[B4] Li P, Zhuo M (1998). Silent glutamatergic synapses and nociception in mammalian spinal cord. Nature.

[B5] Yoshimura M, Jessell T (1990). Amino acid-mediated EPSPs at primary afferent synapses with substantia gelatinosa neurones in the rat spinal cord. J Physiol.

[B6] Li P, Wilding TJ, Kim SJ, Calejesan AA, Huettner JE, Zhuo M (1999). Kainate-receptor-mediated sensory synaptic transmission in mammalian spinal cord. Nature.

[B7] Li P, Kerchner GA, Sala C, Wei F, Huettner JE, Sheng M, Zhuo M (1999). AMPA receptor-PDZ interactions in facilitation of spinal sensory synapses. Nat Neurosci.

[B8] Bardoni R, Magherini PC, MacDermott AB (1998). NMDA EPSCs at glutamatergic synapses in the spinal cord dorsal horn of the postnatal rat. J Neurosci.

[B9] Wang GD, Zhuo M (2002). Synergistic enhancement of glutamate-mediated responses by serotonin and forskolin in adult mouse spinal dorsal horn neurons. J Neurophysiol.

[B10] Zhuo M (2000). Silent glutamatergic synapses and long-term facilitation in spinal dorsal horn neurons. Prog Brain Res.

[B11] Li P, Zhuo M (2001). Substance P and neurokinin A mediate sensory synaptic transmission in young rat dorsal horn neurons. Brain Res Bull.

[B12] Shibata H (1993). Efferent projections from the anterior thalamic nuclei to the cingulate cortex in the rat. J Comp Neurol.

[B13] Yamamura H, Iwata K, Tsuboi Y, Toda K, Kitajima K, Shimizu N, Nomura H, Hibiya J, Fujita S, Sumino R (1996). Morphological and electrophysiological properties of ACCx nociceptive neurons in rats. Brain Res.

[B14] Wang CC, Shyu BC (2004). Differential projections from the mediodorsal and centrolateral thalamic nuclei to the frontal cortex in rats. Brain Res.

[B15] Zhao MG, Toyoda H, Lee YS, Wu LJ, Ko SW, Zhang XH, Jia Y, Shum F, Xu H, Li BM (2005). Roles of NMDA NR2B subtype receptor in prefrontal long-term potentiation and contextual fear memory. Neuron.

[B16] Wu LJ, Toyoda H, Zhao MG, Lee YS, Tang J, Ko SW, Jia YH, Shum FW, Zerbinatti CV, Bu G (2005). Upregulation of forebrain NMDA NR2B receptors contributes to behavioral sensitization after inflammation. J Neurosci.

[B17] Calejesan AA, Kim SJ, Zhuo M (2000). Descending facilitatory modulation of a behavioral nociceptive response by stimulation in the adult rat anterior cingulate cortex. Eur J Pain.

[B18] Zhao MG, Ko SW, Wu LJ, Toyoda H, Xu H, Quan J, Li J, Jia Y, Ren M, Xu ZC (2006). Enhanced presynaptic neurotransmitter release in the anterior cingulate cortex of mice with chronic pain. J Neurosci.

[B19] Lee DE, Kim SJ, Zhuo M (1999). Comparison of behavioral responses to noxious cold and heat in mice. Brain Res.

[B20] Johansen JP, Fields HL, Manning BH (2001). The affective component of pain in rodents: direct evidence for a contribution of the anterior cingulate cortex. Proc Natl Acad Sci USA.

[B21] Zhuo M (2002). Glutamate receptors and persistent pain: targeting forebrain NR2B subunits. Drug Discov Today.

[B22] Talbot JD, Marrett S, Evans AC, Meyer E, Bushnell MC, Duncan GH (1991). Multiple representations of pain in human cerebral cortex. Science.

[B23] Rainville P, Duncan GH, Price DD, Carrier B, Bushnell MC (1997). Pain affect encoded in human anterior cingulate but not somatosensory cortex. Science.

[B24] Casey KL (1999). Forebrain mechanisms of nociception and pain: analysis through imaging. Proc Natl Acad Sci USA.

[B25] Rainville P, Bushnell MC, Duncan GH (2001). Representation of acute and persistent pain in the human CNS: potential implications for chemical intolerance. Ann N Y Acad Sci.

[B26] Koyama T, Kato K, Tanaka YZ, Mikami A (2001). Anterior cingulate activity during pain-avoidance and reward tasks in monkeys. Neurosci Res.

[B27] Eisenberger NI, Lieberman MD, Williams KD (2003). Does rejection hurt? An FMRI study of social exclusion. Science.

[B28] Derbyshire SW, Whalley MG, Stenger VA, Oakley DA (2004). Cerebral activation during hypnotically induced and imagined pain. Neuroimage.

[B29] Johansen JP, Fields HL (2004). Glutamatergic activation of anterior cingulate cortex produces an aversive teaching signal. Nat Neurosci.

[B30] Singer T, Seymour B, O'Doherty J, Kaube H, Dolan RJ, Frith CD (2004). Empathy for pain involves the affective but not sensory components of pain. Science.

[B31] de Tommaso M, Losito L, Difruscolo O, Libro G, Guido M, Livrea P (2005). Changes in cortical processing of pain in chronic migraine. Headache.

[B32] Lei LG, Sun S, Gao YJ, Zhao ZQ, Zhang YQ (2004). NMDA receptors in the anterior cingulate cortex mediate pain-related aversion. Exp Neurol.

[B33] Tang J, Ko S, Ding HK, Qiu CS, Calejesan AA, Zhuo M (2005). Pavlovian fear memory induced by activation in the anterior cingulate cortex. Mol Pain.

[B34] Jasmin L, Rabkin SD, Granato A, Boudah A, Ohara PT (2003). Analgesia and hyperalgesia from GABA-mediated modulation of the cerebral cortex. Nature.

[B35] Woolf CJ, Salter MW (2000). Neuronal plasticity: increasing the gain in pain. Science.

[B36] Willis WD (2002). Long-term potentiation in spinothalamic neurons. Brain Res Brain Res Rev.

[B37] Ji RR, Kohno T, Moore KA, Woolf CJ (2003). Central sensitization and LTP: do pain and memory share similar mechanisms?. Trends Neurosci.

[B38] Zhuo M (2003). Synaptic and molecular mechanisms of glutamatergic synapses in pain and memory. Sheng Li Xue Bao.

[B39] Ikeda H, Heinke B, Ruscheweyh R, Sandkuhler J (2003). Synaptic plasticity in spinal lamina I projection neurons that mediate hyperalgesia. Science.

[B40] Wei F, Vadakkan KI, Toyoda H, Wu LJ, Zhao MG, Xu H, Shum FW, Jia YH, Zhuo M (2006). Calcium calmodulin-stimulated adenylyl cyclases contribute to activation of extracellular signal-regulated kinase in spinal dorsal horn neurons in adult rats and mice. J Neurosci.

[B41] Wei F, Li P, Zhuo M (1999). Loss of synaptic depression in mammalian anterior cingulate cortex after amputation. J Neurosci.

[B42] Wu LJ, Zhao MG, Toyoda H, Ko SW, Zhuo M (2005). Kainate receptor-mediated synaptic transmission in the adult anterior cingulate cortex. J Neurophysiol.

[B43] Wu LJ, Xu H, Ren M, Zhuo M (2007). Genetic and pharmacological studies of GluR5 modulation of inhibitory synaptic transmission in the anterior cingulate cortex of adult mice. Dev Neurobiol.

[B44] Wu LJ, Ko SW, Zhuo M (2007). Kainate receptors and pain: from dorsal root ganglion to the anterior cingulate cortex. Curr Pharm Des.

[B45] Wu LJ, Ko SW, Toyoda H, Zhao MG, Xu H, Vadakkan KI, Ren M, Knifed E, Shum F, Quan J (2007). Increased Anxiety-Like Behavior and Enhanced Synaptic Efficacy in the Amygdala of GluR5 Knockout Mice. PLoS ONE.

[B46] Liauw J, Wu LJ, Zhuo M (2005). Calcium-stimulated adenylyl cyclases required for long-term potentiation in the anterior cingulate cortex. J Neurophysiol.

[B47] Wei F, Xia XM, Tang J, Ao H, Ko S, Liauw J, Qiu CS, Zhuo M (2003). Calmodulin regulates synaptic plasticity in the anterior cingulate cortex and behavioral responses: a microelectroporation study in adult rodents. J Neurosci.

[B48] Xia Z, Storm DR (1997). Calmodulin-regulated adenylyl cyclases and neuromodulation. Curr Opin Neurobiol.

[B49] Wei F, Qiu CS, Kim SJ, Muglia L, Maas JW, Pineda VV, Xu HM, Chen ZF, Storm DR, Muglia LJ (2002). Genetic elimination of behavioral sensitization in mice lacking calmodulin-stimulated adenylyl cyclases. Neuron.

[B50] Toyoda H, Wu LJ, Zhao MG, Xu H, Zhuo M (2007). Time-dependent postsynaptic AMPA GluR1 receptor recruitment in the cingulate synaptic potentiation. Dev Neurobiol.

[B51] Hayashi Y, Shi SH, Esteban JA, Piccini A, Poncer JC, Malinow R (2000). Driving AMPA receptors into synapses by LTP and CaMKII: requirement for GluR1 and PDZ domain interaction. Science.

[B52] Passafaro M, Piech V, Sheng M (2001). Subunit-specific temporal and spatial patterns of AMPA receptor exocytosis in hippocampal neurons. Nat Neurosci.

[B53] Wei F, Qiu CS, Liauw J, Robinson DA, Ho N, Chatila T, Zhuo M (2002). Calcium calmodulin-dependent protein kinase IV is required for fear memory. Nat Neurosci.

[B54] Escobar ML, Chao V, Bermudez-Rattoni F (1998). In vivo long-term potentiation in the insular cortex: NMDA receptor dependence. Brain Res.

[B55] Wei F, Zhuo M (2001). Potentiation of sensory responses in the anterior cingulate cortex following digit amputation in the anaesthetised rat. J Physiol.

[B56] Wu MF, Pang ZP, Zhuo M, Xu ZC (2005). Prolonged membrane potential depolarization in cingulate pyramidal cells after digit amputation in adult rats. Mol Pain.

[B57] Wei F, Wang GD, Kerchner GA, Kim SJ, Xu HM, Chen ZF, Zhuo M (2001). Genetic enhancement of inflammatory pain by forebrain NR2B overexpression. Nat Neurosci.

[B58] Zhuo M, Gebhart GF (1990). Spinal cholinergic and monoaminergic receptors mediate descending inhibition from the nuclei reticularis gigantocellularis and gigantocellularis pars alpha in the rat. Brain Res.

[B59] Zhuo M, Gebhart GF (1990). Characterization of descending inhibition and facilitation from the nuclei reticularis gigantocellularis and gigantocellularis pars alpha in the rat. Pain.

[B60] Zhuo M, Gebhart GF (1991). Spinal serotonin receptors mediate descending facilitation of a nociceptive reflex from the nuclei reticularis gigantocellularis and gigantocellularis pars alpha in the rat. Brain Res.

[B61] Zhuo M, Gebhart GF (1992). Characterization of descending facilitation and inhibition of spinal nociceptive transmission from the nuclei reticularis gigantocellularis and gigantocellularis pars alpha in the rat. J Neurophysiol.

[B62] Zhuo M, Gebhart GF (1997). Biphasic modulation of spinal nociceptive transmission from the medullary raphe nuclei in the rat. J Neurophysiol.

[B63] Zhuo M, Sengupta JN, Gebhart GF (2002). Biphasic modulation of spinal visceral nociceptive transmission from the rostroventral medial medulla in the rat. J Neurophysiol.

[B64] Zhuo M, Gebhart GF (2002). Facilitation and attenuation of a visceral nociceptive reflex from the rostroventral medulla in the rat. Gastroenterology.

[B65] Calejesan AA, Ch'ang MH, Zhuo M (1998). Spinal serotonergic receptors mediate facilitation of a nociceptive reflex by subcutaneous formalin injection into the hindpaw in rats. Brain Res.

[B66] Robinson DA, Wei F, Wang GD, Li P, Kim SJ, Vogt SK, Muglia LJ, Zhuo M (2002). Oxytocin mediates stress-induced analgesia in adult mice. J Physiol.

[B67] Urban MO, Gebhart GF (1999). Supraspinal contributions to hyperalgesia. Proc Natl Acad Sci USA.

[B68] Porreca F, Ossipov MH, Gebhart GF (2002). Chronic pain and medullary descending facilitation. Trends Neurosci.

[B69] Robinson DA, Calejesan AA, Wei F, Gebhart GF, Zhuo M (2004). Endogenous facilitation: from molecular mechanisms to persistent pain. Curr Neurovasc Res.

[B70] Coull JA, Beggs S, Boudreau D, Boivin D, Tsuda M, Inoue K, Gravel C, Salter MW, De Koninck Y (2005). BDNF from microglia causes the shift in neuronal anion gradient underlying neuropathic pain. Nature.

[B71] Tsuda M, Shigemoto-Mogami Y, Koizumi S, Mizokoshi A, Kohsaka S, Salter MW, Inoue K (2003). P2X4 receptors induced in spinal microglia gate tactile allodynia after nerve injury. Nature.

